# Supplementary material for: Rapid calcification propensity testing in blood using a temperature controlled microfluidic polymer chip
Source: PLoS One. 2020 Apr 7;15(4):e0230493. doi: 10.1371/journal.pone.0230493 (PMC7138308; doi:10.1371/journal.pone.0230493)
Supplement: Attachment — Submitted filename: Response to Reviewers- 19-22409.docx [file pone.0230493.s001.docx]

The work builds

6 on two previous manuscripts “Quick test of calcification propensity in polymer microchips”,

7 (PONE-D-18-29628 and PONE-D-19-17271), which we retracted after receiving the

8 reviewers’ comments. We felt that the comments reflected serious misunderstandings

9 about the nature of our publication, and that we needed more time than the review process

10 would allow us, to address all issues. We should like to point out that publishing in the

11 Engineering world and publishing in the Biomedical world are vastly different exercises. Our

12 first version manuscripts were more proper for the Engineering community. However, we

13 specifically want to address a much more general readership with this current manuscript.

14 Therefor, we had to enhance, reformat and elaborate on the work. We hope that the

15 manuscript is now intelligible by a wide readership and can be published in PLoS ONE, which

16 we feel is the perfect platform for publishing such cross-disciplinary work including

17 engineering, biochemistry and clinical routine. Our work is specifically NOT a clinical study,

18 but a proof-of-concept study on how to substantially improve a functional clinical assay,

19 which has proved useful in previous studies, but just takes too long.

20

21 We have substantially enhanced the contents of the manuscript and therefore submit de

22 novo, but we gladly oblige your request to answer point-by-point to the concerns raised by

23 the reviewers at the time. Reflecting the substantial changes made to the manuscript, we

24 decided to shift the corresponding authorship to Professor Jahnen-Dechent.

25

26 Our answers to your questions are typed in italics.

27

28 1. Please ensure that your manuscript meets PLOS ONE's style requirements, including those

29 for file naming. The PLOS ONE style templates can be found at

30 http://www.journals.plos.org/plosone/s/file?id=wjVg/PLOSOne_formatting_sample_main_b

31 ody.pdf andhttp://www.journals.plos.org/plosone/s/file?id=ba62/PLOSOne_formatting_sam

32 ple_title_authors_affiliations.pdf

33

34 *The manuscript is now formatted exactly following PLoS ONE style outlined in the above*

35 *mentioned documents.*

36

37 2. Thank you for stating the following in the Financial Disclosure section:

38

39 "FEDERAL MINISTRY FOR ECONOMIC AFFAIRS AND ENERGY - ZIM under grant number

40 ZF4429601AJ7

41

42 https://www.zim.de/ZIM/Navigation/DE/Home/home.html

43

44 The funders had no role in study design, data collection and analysis, decision to publish, or

45 preparation of the manuscript.

46

47 We note that one or more of the authors are employed by a commercial company: Calciscon

48 AG

Cover Letter

49

50 a. Please provide an amended Funding Statement declaring this commercial affiliation, as

51 well as a statement regarding the Role of Funders in your study. If the funding organization

52 did not play a role in the study design, data collection and analysis, decision to publish, or

53 preparation of the manuscript and only provided financial support in the form of authors'

54 salaries and/or research materials, please review your statements relating to the author

55 contributions, and ensure you have specifically and accurately indicated the role(s) that

56 these authors had in your study. You can update author roles in the Author Contributions

57 section of the online submission form.

58

59 Please also include the following statement within your amended Funding Statement.

60 “The funder provided support in the form of salaries for authors [insert relevant initials], but

61 did not have any additional role in the study design, data collection and analysis, decision to

62 publish, or preparation of the manuscript. The specific roles of these authors are articulated

63 in the ‘author contributions’ section.”

64

65 If your commercial affiliation did play a role in your study, please state and explain this role

66 within your updated Funding Statement.

67

68 b. Please also provide an updated Competing Interests Statement declaring this commercial

69 affiliation along with any other relevant declarations relating to employment, consultancy,

70 patents, products in development, or marketed products, etc

71

72 Within your Competing Interests Statement, please confirm that this commercial affiliation

73 does not alter your adherence to all PLOS ONE policies on sharing data and materials by

74 including the following statement: "This does not alter our adherence to PLOS ONE policies

75 on sharing data and materials.” (as detailed online in our guide for

76 authors http://journals.plos.org/plosone/s/competing-interests) . If this adherence

77 statement is not accurate and there are restrictions on sharing of data and/or materials,

78 please state these. Please note that we cannot proceed with consideration of your article

79 until this information has been declared.

80

81 c. Please include both an updated Funding Statement and Competing Interests Statement in

82 your cover letter. We will change the online submission form on your behalf.

83

84 Please know it is PLOS ONE policy for corresponding authors to declare, on behalf of all

85 authors, all potential competing interests for the purposes of transparency. PLOS defines a

86 competing interest as anything that interferes with, or could reasonably be perceived as

87 interfering with, the full and objective presentation, peer review, editorial decision-making,

88 or publication of research or non-research articles submitted to one of the journals.

89 Competing interests can be financial or non-financial, professional, or personal. Competing

90 interests can arise in relationship to an organization or another person. Please follow this

91 link to our website for more details on competing

92 interests:http://journals.plos.org/plosone/s/competing-interests

93

94 *This work was supported by a fund of the Federal Ministry for economic affairs and energy of*

95 *the Federal Republic of Germany (https://www.bmwi.de/Redaktion/EN/Artikel/SME*96

*Sector/technology-neutral-project-support-01.html; grant number ZIM ZF4429601AJ7)*

*awarded to WKS and by grants awarded to WJD by the IZKF Aachen of* 97 *the Medical Faculty of*

98 *RWTH Aachen (https://www.izkf.rwth-aachen.de/index.php/en/izkf-aachen) and by the*

99 *German Research Foundation (https://www.dfg.de/en/index.jsp; DFG SFB/TRR219-Project C-*

100 *03). The funders had no role in study design, data collection and analysis, decision to publish,*

101 *or preparation of the manuscript. AP and WJD are co-founders and share holders of Calciscon*

102 *AG, a start-up company based at Nidau, Switzerland. AP is an employee of Calciscon AG, who*

103 *provided support in the form of salaries for authors AP, but did not have any additional role*

104 *in the study design, data collection and analysis, decision to publish, or preparation of the*

105 *manuscript. The commercial affiliation does not alter our adherence to PLoS ONE policies on*

106 *sharing data and materials.*

107

108 3. We note that Figure 1 in your submission contains copyrighted images.

109

110 All PLOS content is published under the Creative Commons Attribution License (CC BY 4.0),

111 which means that the manuscript, images, and Supporting Information files will be freely

112 available online, and any third party is permitted to access, download, copy, distribute, and

113 use these materials in any way, even commercially, with proper attribution. For more

114 information, see our copyright guidelines:http://journals.plos.org/plosone/s/licenses-and115

copyright.

116

117 We require you to either (a) present written permission from the copyright holder to publish

118 this figure specifically under the CC BY 4.0 license, or (b) remove the figure from your

119 submission:

120

121 a. You may seek permission from the original copyright holder of Figure 1 to publish the

122 content specifically under the CC BY 4.0 license.

123

124 We recommend that you contact the original copyright holder with the Content Permission

125 Form (http://journals.plos.org/plosone/s/file?id=7c09/content-permission-form.pdf) and the

126 following text:

127 “I request permission for the open-access journal PLOS ONE to publish XXX under the

128 Creative Commons Attribution License (CCAL) CC BY 4.0

129 (http://creativecommons.org/licenses/by/4.0/). Please be aware that this license allows

130 unrestricted use and distribution, even commercially, by third parties. Please reply and

131 provide explicit written permission to publish XXX under a CC BY license and complete the

132 attached form.”

133

134 Please upload the completed Content Permission Form or other proof of granted

135 permissions as an "Other" file with your submission

136

137 In the figure caption of the copyrighted figure, please include the following text: “Reprinted

138 from [ref] under a CC BY license, with permission from [name of publisher], original

139 copyright [original copyright year].”

140

141 b. If you are unable to obtain permission from the original copyright holder to publish

142 these figures under the CC BY 4.0 license or if the copyright holder’s requirements are

143 incompatible with the CC BY 4.0 license, please either i) remove the figure or ii) supply a

144 replacement figure that complies with the CC BY 4.0 license. Please check copyright

information on all replacement figures and update 145 the figure caption with source

146 information. If applicable, please specify in the figure caption text when a figure is similar

147 but not identical to the original image and is therefore for illustrative purposes only.

148

149 *We apologize for this annoying lapsus. We present a replacement version of Fig 1 without*

150 *copyright protection. In any case, as you surmised, the figure is for illustrative purpose only.*

151

152 [Note: HTML markup is below. Please do not edit.]

153

154 Reviewers' comments:

155

156 Reviewer's Responses to Questions

157

158 **Comments to the Author**

159

160 1. Is the manuscript technically sound, and do the data support the conclusions?

161

162 The manuscript must describe a technically sound piece of scientific research with data that

163 supports the conclusions. Experiments must have been conducted rigorously, with

164 appropriate controls, replication, and sample sizes. The conclusions must be drawn

165 appropriately based on the data presented

166

167 Reviewer #2: Yes

168 We thank reviewer 1 for this supportive statement.

169 Reviewer #3: No

170 We hope that the revised version of our manuscript now convinces reviewer 3 that the work

171 is technically sound.

172

173

174 2. Has the statistical analysis been performed appropriately and rigorously?

175

176 Reviewer #2: Yes

177

178 Reviewer #3: N/A

179

180

181

182 3. Have the authors made all data underlying the findings in their manuscript fully available?

183

184 The PLOS Data policy requires authors to make all data underlying the findings described in

185 their manuscript fully available without restriction, with rare exception (please refer to the

186 Data Availability Statement in the manuscript PDF file). The data should be provided as part

187 of the manuscript or its supporting information, or deposited to a public repository. For

188 example, in addition to summary statistics, the data points behind means, medians and

189 variance measures should be available. If there are restrictions on publicly sharing data—e.g.

190 participant privacy or use of data from a third party—those must be specified.

191

192 Reviewer #2: Yes

193

194 Reviewer #3: No

195

196 *All data pertinent to this manuscript are in fact contained. Therefor we respectfully disagree*

197 *with reviewer #3 on this point.*

198

199

200 4. Is the manuscript presented in an intelligible fashion and written in standard English?

201

202 PLOS ONE does not copyedit accepted manuscripts, so the language in submitted articles

203 must be clear, correct, and unambiguous. Any typographical or grammatical errors should be

204 corrected at revision, so please note any specific errors here.

205

206 Reviewer #2: Yes

207

208 Reviewer #3: No

209

210 *We have reworded substantial parts oft he manuscript to improve reading. The authors have*

211 *previously published in high profile journals listed in the reference section. No concerns were*

212 *ever raised about language errors. Therefor we kindly ask reviewer 3 to specify exactly what*

213 *he or she sees unfit for publication and we will gladly amend.*

214

215

216 5. Review Comments to the Author

217

218 Please use the space provided to explain your answers to the questions above. You may also

219 include additional comments for the author, including concerns about dual publication,

220 research ethics, or publication ethics. (Please upload your review as an attachment if it

221 exceeds 20,000 characters)

222

223 Reviewer #2: The authors present an improved system to measure calcification propensity

224 which enables a 36-faster measurement.

225 Figure 5: no linear relationship: any explanation? (to be incleded within the discussion).

226 *The thank the reviewer for raising this point. We have included an explanation in the*

227 *discussion:*

228 *“The T50 acceleration was not linear, which might have been expected based on previous*

229 *time-resolved small-angle X-ray and quantitative small-angle neutron scattering studies*

230 *indicated first order CPP formation kinetics [38-40] at 37 °C. We hypothesize that CPP*

231 *formation and transformation of primary into secondary CPP at elevated temperature are*

232 *more complex, because formation and dissolution of primary CPP, pH stability of the buffer*

233 *system, and thermal denaturation of serum proteins will all influence the overall T50 time*

234 *apart form the solute concentrations. These points require further physicochemical studies*

235 *beyond the scope of this work. Nevertheless, we observed close correlation of test*

236 *temperature and T50 time up to 75 °C. ….. ff.”*

237

238 Figure 6: blue area healthy individuals: how is this area defined?

239 *The blue area in the original figure represented the T50 range determined in sera from*

240 *healthy 253 Swiss adults by the Nephelometer-based test. Reflecting the reviewer’s*

*statement we noticed that the blue range strictly* 241 *only represented Nephelometer-derived*

242 *T50 values and thus the lower part of the graph, but not the chip-derived T50 values.*

243 *Therefor we deleted the blue square and state:*

244 *“The measuring range presented in Fig 7 covered previously published nephelometer-based*

245 *T50 values including a reference range of healthy subjects (270 to 460 minutes), determined*

246 *in 253 Swiss adults [24], values determined in 184 patients with CKD stages 3 to 4 (mean, 329*

247 *± 95 minutes) [23] and in 2,785 patients undergoing hemodialysis (median, 212 [10th-90th*

248 *percentiles, 109-328] minutes) [25].”*

249

250 Discussion needs to be extended.

251 It should be clearly stated that the data are preliminary and – in future - need extension in

252 more samples and larger cohorts and validation for risk prediction.

253 Moreover, potential technical problems should be addressed i.e. protein denaturation in

254 more detail or potential problems throughout the manufacturing process.

255 *We thank the reviewer for these valid and helpful comments. We addressed all points raised*

256 *in the new manuscript and are happy to oblige should the reviewer suggest further changes.*

257

258 Minor:

259 Figure 3: abbrevation LDR

260

261 Reviewer #3: In this paper, Bavendiek and colleagues describe a test system to measure

262 calcification propensity in serum samples using a custom-made microchip with turbidity

263 detection. The peculiarity of this test system is that it can be operated at much higher

264 temperatures than a nephelometer, thereby reducing the analysis time to a few minutes

265 compared to hours.

266 The fabrication of the microchip and the analysis conditions are reported in detail. The

267 turbidity reaction was measured at supernormal calcium and phosphate concentrations of

268 28 and 24 mmol/L, respectively. The temperature dependence of the reaction is shown over

269 a range of 60-75 degrees Celsius using one serum sample. At higher temperature, the half270

time of the reaction (T50) was shorter and turbidity at the end of the reaction appeared to

271 be higher compared with lower temperatures.

272 In a second experiment, 2 serum samples from healthy individuals and 3 samples from

273 patients were analyzed with the microchip at 75 degrees Celsius in comparison with a

274 standard nephelometric device showing a linear correlation of results between the two

275 analysis methods.

276 *We thank the reviewer for this valid representation of the work presented in PONE-D-18-*

277 *29628. We trust that this current manuscript is much improved both in content and*

278 *presentation, and addresses all points raised by the reviewers.*

279

280 Main comments

281 Microchips are certainly a very innovative means of future test systems. Measurement of

282 calcification propensity is a relevant issue in patients with chronic kidney disease mineral

283 and bone disorders (CKD-MBD).

284 However, the submitted work is for the most part a repetition of a recently published work

285 of the authors. This refers to the Figures 1, 2, 4 and 5. Only Figure 3 and 6 with results on

286 five serum samples are new.

287

*We have substantially elaborated on both the chip design, manufacture* 288 *and the proof-of*289

*concept study. All figures were modified to qualify as “new”. A pre-publication manuscript of*

290 *this work was deposited as a printed abstract of the Biomedical Technology Conference*

291 *BMT2018 (ref 44). Like all other printed abstracts of this conference, this contribution*

292 *underwent formal screening by the conference chairs, but not full peer review and therefore*

293 *cannot be counted as a published paper. I know for a fact, because I (WJD) was one of the*

294 *conference organizers. We have added a statement in the acknowledgements to that effect.*

295

296 The 5 individuals included in this analysis are not described in any detail (total protein,

297 albumin and fetuin concentration, concentration of other minerals in the samples). How is

298 ‘high propensity of calcification’ in the 3 dialysis patients defined, had these patients

299 clinically recognizable calcifications?

300 *This current work represents a proof-of-concept study, not a clinical study. Therefor it suffices*

301 *to measure a range of authentic samples that have been previously measured in other*

302 *studies using the Nephelometer-based T50 test. No correlation with any disease whatsoever*

303 *is made based on the chip-derived T50 values at this point, except that the T50 values*

304 *correlate very well with the established Nephelometer based T50 test. We agree with*

305 *reviewer 3 that a full clinical study employing this novel test must and will include all relevant*

306 *patient data, like all our previous clinical studies did. Some, but not all of these studies and*

307 *similar studies from other research labs are cited.*

308

309 At higher temperature protein denaturation could occur and interfere with the turbidity

310 measurement of calcium phosphate particle formation. Protein denaturation needs to

311 excluded by appropriate experiments.

312 *We thank reviewer for raising this important point, which we now address in the discussion.*

313 *We write:*

314 *“We hypothesize that CPP formation and transformation of primary into secondary CPP at*

315 *elevated temperature are more complex, because formation and dissolution of primary CPP,*

316 *pH stability of the buffer system, and thermal denaturation of serum proteins will all*

317 *influence the overall T50 time apart form the solute concentrations. These points require*

318 *further physicochemical studies beyond the scope of this work. Nevertheless, we observed*

319 *close correlation of test temperature and T50 time up to 75 °C. ….. ff.”*

320

321 Also, experiments showing that different concentrations of total protein, albumin, and fetuin

322 affect calcium phosphate particle formation in this test system would be helpful, e.g. by

323 adding defined amounts of proteins to samples.

324 *This will require further studies beyond the scope of this proof-of-concept study as mentioned*

325 *in the discussion.*

326

327 Minor points

328 Abstract, line 4: ‘patients suffering from a kidney with insufficient performance’ is an

329 unusual term; ‘patients with CKD-MBD’ may be more appropriate.

330 We now use CKD or ESRD, because most patients in this study are dialysis patients. Whether

331 or not they have MBD, i.e. compromized bone metabolism, was not studied.

332 Abstract, line 8: ‘conversion process’ is not understandable in this context and needs

333 explanation.

334

335

336 Experimental setup:

337 The choice of supernormal calcium and phosphate concentration should be justified. How do

338 these concentrations relate to the physiological setting and morbidity?

339 *“Supernormal” calcium and phosphate concentration are the main drivers of the T50 test and*

340 *therefor instrumental. The buffer system and the ratios of serum and mineral was optimized*

341 *to reliably drive CPP formation and transformation, yet to avoid the stochastic mineralization*

342 *observed at low supersaturation. The whole setup was described in great detail in the*

343 *original publication including a comprehensive supplement. This stringent description in fact*

344 *warranted worldwide patents. We refer the reviewer to this publication, that has since*

345 *proven tremendously useful in many clinical studies from several laboratories relating to*

346 *various physiological settings, morbidity and mortality. Some of that literature is now cited.*

347

348 Apparently, the measurement is done under flow conditions. These should be explained in

349 more detail.

350 *Apart from filling the cartridge with mixed reaction solution there was no flow during the*

351 *measurements. We apologize if this was not clear in the original manuscript. We rephrased*

352 *for clarity.*

353

354 In general the language should be improved

355 *We have reworded substantial parts oft he manuscript to improve reading. The authors have*

356 *previously published in high profile journals listed in the reference section. No concerns were*

357 *ever raised about language errors. Therefor we kindly ask reviewer 3 to specify exactly what*

358 *he or she sees unfit for publication and we will gladly amend.*

359

360 *on behalf of all authors*

361 *Willi Jahnen-Dechent, PhD*
