# Supplementary material for: Rapid calcification propensity testing in blood using a temperature controlled microfluidic polymer chip
Source: PLoS One. 2020 Apr 7;15(4):e0230493. doi: 10.1371/journal.pone.0230493 (PMC7138308; doi:10.1371/journal.pone.0230493)
Supplement: Attachment — Submitted filename: Response To Reviewers.docx [file pone.0230493.s002.docx]

Response To Reviewers

Our answer: We would like to thank all reviewers for their time and effort to improve the quality of our manuscript. We respond to their comments point-by-point.

Reviewers' comments:

Reviewer's Responses to Questions

Comments to the Author

1. Is the manuscript technically sound, and do the data support the conclusions?

Reviewer #1: Yes

Reviewer #2: Yes

2. Has the statistical analysis been performed appropriately and rigorously?

Reviewer #1: Yes

Reviewer #2: Yes

3. Have the authors made all data underlying the findings in their manuscript fully available?

Reviewer #1: Yes

Reviewer #2: Yes

4. Is the manuscript presented in an intelligible fashion and written in standard English?

Reviewer #1: Yes

Reviewer #2: No

5. Review Comments to the Author

Reviewer #1: In this manuscript, Bavendiek et al. present a newly designed microfluidic polymer chip which reduces times for measuring calcification propensity and may form a basis for future point of care tests. The most important part of this test is that measurement of calcification propensity can be achieved at much higher temperatures than the previous test thereby reducing measurement time from 600 to just a few minutes.

It is a engineering-orientated paper with detailed reports on the fabrication and optimization of the microchip.

Our answer: We thank reviewer 1 for their appreciation of our work and the suggestions made in the comments.

Main comments:

- What would be the benefit of a possible bedside test? Would there be changes in conducting dialysis or is calcification propensity rather a long-term parameter?

Our answer: The benefit of a bedside, or better still, an in-line test could be patient individualized dialysis. An example may be taken from work cited in ref. 30 showing the “The Effect of Increasing Dialysate Magnesium on Serum Calcification Propensity in Subjects with End Stage Kidney Disease”. While such patient-individualized dialysis may still seem a long way off, an improved T50 test could be an important first step, because it better reflects the phosphorous woes (see ref 17) comprising phosphate, serum protein concentration, magnesium, pyrophosphate etc. than does serum phosphate alone.

- The introduction does not match the actual paper. Introduction handles kidney disease, hyperphosphatemia, morbidity, dialysis, clinical outcome etc, but the paper is an engineering-based approach. This should be adjusted to either more data on clinical use (e.g. adding a small clinical trial) or going all the way to an engineering paper.

Our answer: We thank the reviewer for this suggestion. In the introduction we toned done on clinical findings and focused on the task at hand, technical improvement of the T50 assay.

- The sera of 5 patients was used for validation of the test. But there is no data on the used test sera (Figure 7). Did the patients suffer from clinically relevant calcification? CAD?

Our answer: We have no information on the health status of the donors, because the samples were anonymized. To perform the repeat measurements shown in Fig 6 (was Fig 7) anonymized serum samples were blended to cover the range of published T50 studies, and to obtain sufficient amount of serum for repeat measurements. For clinical information we refer reviewer to the clinical studies employing the original T50 assay, some of which are cited.

- Figure 1 seems to be redundant.

Our answer: we deleted Fig 1.

Reviewer #2: Bavendiek et al demonstrated an improved temperature controlled T50 test format which reports Overall calcification propensity in serum.

While this is an important topic, there are some major limitations of this manuscript which might it challenging to follow. The authors should more emphasize the aim of the study, shorten the introduction and extend the discussion.

Our answer: We thank the reviewer for this suggestion. In the introduction we toned done on clinical findings and focused on the task at hand, technical improvement of the T50 assay. At this stage we would like to refrain from further discussion of this proof-of-principle study until we have completed a first clinical study using the novel chip-based T50 assay. We hope the reviewers can agree with this.
