# Supplementary material for: Rapid calcification propensity testing in blood using a temperature controlled microfluidic polymer chip
Source: PLoS One. 2020 Apr 7;15(4):e0230493. doi: 10.1371/journal.pone.0230493 (PMC7138308; doi:10.1371/journal.pone.0230493)
Supplement: Attachment — Submitted filename: Response To Reviewers R2.docx [file pone.0230493.s003.docx]

PONE-D-19-22409R1

Rapid calcification propensity testing in blood using a temperature controlled microfluidic polymer chip

PLOS ONE

Dear Prof Marx,

Thank you again for handling our manuscript. We are naturally pleased that minor revisions may render our manuscript suitable for publication. We thank both reviewers for their continued help and favorable judgement including their suggestion to comment on the potential clinical impact of this chip. We are happy to oblige. As before, we comment point-by-point quoting the original text. We hope the manuscript now meets the publication criteria and we look forward to your reply.

Kind regards

Willi Jahnen-Dechent, on behalf of the authors

PONE-D-19-22409R1
Rapid calcification propensity testing in blood using a temperature controlled microfluidic polymer chip
PLOS ONE

Dear Prof Jahnen-Dechent,

Thank you for submitting your manuscript to PLOS ONE. After careful consideration, we feel that it has merit but does not fully meet PLOS ONE’s publication criteria as it currently stands. Therefore, we invite you to submit a revised version of the manuscript that addresses the points raised during the review process and quoted below.

We would appreciate receiving your revised manuscript by Mar 12 2020 11:59PM. When you are ready to submit your revision, log on to <https://www.editorialmanager.com/pone/> and select the 'Submissions Needing Revision' folder to locate your manuscript file.

If you would like to make changes to your financial disclosure, please include your updated statement in your cover letter.

To enhance the reproducibility of your results, we recommend that if applicable you deposit your laboratory protocols in [protocols.io](http://protocols.io), where a protocol can be assigned its own identifier (DOI) such that it can be cited independently in the future. For instructions see: <http://journals.plos.org/plosone/s/submission-guidelines#loc-laboratory-protocols>

Please include the following items when submitting your revised manuscript:

- A rebuttal letter that responds to each point raised by the academic editor and reviewer(s). This letter should be uploaded as separate file and labeled 'Response to Reviewers'.
- A marked-up copy of your manuscript that highlights changes made to the original version. This file should be uploaded as separate file and labeled 'Revised Manuscript with Track Changes'.
- An unmarked version of your revised paper without tracked changes. This file should be uploaded as separate file and labeled 'Manuscript'.

Please note while forming your response, if your article is accepted, you may have the opportunity to make the peer review history publicly available. The record will include editor decision letters (with reviews) and your responses to reviewer comments. If eligible, we will contact you to opt in or out.

We look forward to receiving your revised manuscript.

Kind regards,

Marc W. Merx, MD
Academic Editor
PLOS ONE


Reviewers' comments:

Reviewer's Responses to Questions

**Comments to the Author**

6. Review Comments to the Author

Please use the space provided to explain your answers to the questions above. You may also include additional comments for the author, including concerns about dual publication, research ethics, or publication ethics. (Please upload your review as an attachment if it exceeds 20,000 characters)

Reviewer #1: The authors present their revised manuscript on a newly designed microfluidic polymer chip which reduces times for measuring calcification propensity and may form a basis for future in-line tests. The most important part of this test is that measurement of calcification propensity can be achieved at much higher temperatures than the previous test thereby reducing measurement time from 600 to just a few minutes. It is an engineering-orientated paper with detailed reports on the fabrication and optimization of the microchip.
The manuscript has significantly improved due to the changes made in the introduction part.

Main comments:
- We suggest to include one sentence/paragraph on the impact of reduced CPP measurements on dialysis quality in discussion.

Our comment: Following reviewer’s suggestion we added a short paragraph to the discussion

“*Short analysis times are advantageous in clinical settings when T50 measurements inform about the completeness of dialysis or about the influence of varying dialysis protocols or fluids. Importantly, the T50 times reflect the combined calcification propensity including solutes, activators and inhibitors as well as CPP, which might serve as mineralization nuclei that are poorly reflected by conventional phosphate monitoring. Recently, CPP and their precursors were directly end elegantly measured by two independent methods [6, 34]. These and similar methods may further refine the assessment of calcification propensity if miniaturized and accelerated like the chip-based T50 assay presented here.”*

Reviewer #2: In the revised manuscript the authors have adequately addressed the reviewers' comments. I would recommend to add one phrase about the expected clinical benefit/ relevance into the abstract.

Our comment: We added a sentence to the abstract

“The speed and reproducibility of the T50 chip-based assay run at 75°C suggest that it may be suitable for rapid measurements, preferably in-line in a dialyser or in a portable microfluidic analytic device with the chip inserted as a disposable cartridge.”
